# Risk Prediction Model for Taxane-Induced Peripheral Neuropathy in Early-Stage Cancer

**DOI:** 10.1001/jamanetworkopen.2026.4901

**Published:** 2026-04-10

**Authors:** Meghna S. Trivedi, Joseph M. Unger, N. Lynn Henry, Amy K. Darke, Daniel L. Hertz, Thomas H. Brannagan, Stephanie J. Reyes, Bryan P. Schneider, William J. Irvin, Amanda R. Hathaway, Amy C. Vander Woude, Vinay K. Gudena, Paula Cabrera-Galeana, Mary Orsted, Michael LeBlanc, Michael J. Fisch, Dawn L. Hershman

**Affiliations:** 1 Herbert Irving Comprehensive Cancer Center, Columbia University Irving Medical Center, New York, New York; 2 SWOG Cancer Research Network Statistics and Data Management Center, Seattle, Washington; 3 Fred Hutch Cancer Center, Seattle, Washington; 4 Department of Internal Medicine, University of Michigan Medical School, Ann Arbor; 5 Department of Clinical Pharmacy, University of Michigan College of Pharmacy, Ann Arbor; 6 Peripheral Neuropathy Center, Columbia University Medical Center, New York, New York; 7 Nancy N. and J. C. Lewis Cancer and Research Pavilion at St Joseph’s/Candler Oncology Clinical Research, Savannah, Georgia; 8 Department of Medicine, Division of Hematology/Oncology, Indiana University School of Medicine, Indianapolis; 9 Bon Secours Saint Francis Medical Center Cancer Institute/Southeast Clinical Oncology Research (SCOR), Midlothian, Virginia; 10 University Cancer & Blood LLC, Athens, Georgia; 11 The Cancer & Hematology Centers, Grand Rapids, Michigan; 12 Cone Health Cancer Center, Greensboro, North Carolina; 13 Division of Oncology and Hematology, Instituto Nacional de Cancerología, México City, Mexico; 14 HealthPartners Cancer Center at Regions Hospital, St Paul, Minnesota; 15 Department of General Oncology, MD Anderson Cancer Center, Houston, Texas; 16 Specialty Care Solutions, Carelon Medical Benefits Management, Houston, Texas

## Abstract

**Importance:**

Taxane-induced peripheral neuropathy (TIPN) affects quality of life and ability to complete cancer treatment and has limited effective interventions for prevention and treatment.

**Objective:**

To develop and validate a TIPN risk prediction model.

**Design, Setting, and Participants:**

SWOG S1714 was a prospective observational cohort study conducted at sites in the National Cancer Institute National Community Oncology Research Program between March 1, 2019, and November 15, 2021, with 3 years of follow-up. The study included evaluable participants 18 years or older with stage I to III lung, breast, or ovarian, fallopian tube, or primary peritoneal cancer who were starting taxane-based treatment. Statistical analysis was conducted from December 2023 to June 2024.

**Exposures:**

Taxane-based regimens including paclitaxel or docetaxel.

**Main Outcomes and Measures:**

The primary end point was occurrence of TIPN by 24 weeks. TIPN was assessed using the patient-reported European Organization of Research and Treatment of Cancer Quality of Life Questionnaire–Chemotherapy-Induced Peripheral Neuropathy 20-item scale (CIPN-20) at baseline and weeks 4, 8, 12, and 24. Occurrence of TIPN was defined as an increase of 8 points or more over baseline in the CIPN-20 sensory subscale score. With a 60% random sample of evaluable participants, best-subset selection using logistic regression and *k*-fold cross-validation identified a best model based on demographic factors, baseline comorbid conditions, and treatment factors. Adverse risk factors were summed, generating a score, split at the median and tested in the remaining 40% of evaluable participants. The target difference was 12% between high-risk vs low-risk groups.

**Results:**

A total of 1336 participants enrolled in S1714. Of 1278 evaluable participants (median age, 55.0 years [range, 23.0-84.0 years]; 1264 women [98.9%]; 1164 with breast cancer [91.1%]), 804 (62.9%) experienced TIPN by week 24. Using the training set of 768 participants, a risk prediction model for TIPN was developed that included 5 adverse risk factors: receipt of paclitaxel; stage II or III disease; planned taxane duration of more than 12 weeks; diabetes, autoimmune disease, moderate kidney disease, or a neurologic condition; and self-identified race and ethnicity (Black, Hispanic, Native American, Pacific Islander, multiple races, or unknown race or ethnicity). In the test set of 510 participants, TIPN was more common in high-risk (235 of 345 [68.1%]) vs low-risk (84 of 165 [50.9%]) groups (absolute difference, 17.2%), exceeding the 12% target.

**Conclusions and Relevance:**

In this cohort study of participants with early-stage cancer receiving a taxane regimen, a set of baseline risk factors stratified TIPN risk. A risk prediction model may guide treatment decision-making, symptom monitoring, and enrollment in interventional trials for TIPN prevention and treatment.

## Key Points

**Question:**

What risk factors are associated with taxane-induced peripheral neuropathy (TIPN)?

**Findings:**

In this cohort study of 1278 participants with early-stage cancer receiving a taxane-based chemotherapy regimen, a 2-step training and test approach was used to derive a TIPN risk prediction model. A model with 5 adverse risk factors predicted disparate levels of TIPN risk.

**Meaning:**

This study suggests that a risk prediction model for TIPN may guide treatment decision-making, symptom monitoring, and supportive care.

## Teaser

This cohort study develops and validates a risk prediction model for taxane-induced peripheral neuropathy among patients with early-stage cancer.

## Introduction

Taxanes are frequently used in the treatment of breast, gynecologic, and non–small cell lung cancer but may cause taxane-induced peripheral neuropathy (TIPN). TIPN occurs in up to 70% of patients.^1,2,3,4^ There are limited effective interventions for the prevention and treatment of TIPN. The mainstay of management of TIPN during treatment is taxane dose modification.^5^ Dose reductions may adversely affect long-term outcomes such as overall survival.^6,7,8^

Numerous patient-related and treatment-related risk factors for the development of TIPN have been identified. Patient-related risk factors include demographic factors (race and ethnicity^9^ and age^10^), lifestyle factors (body mass index,^11,12^ physical activity,^11^ and smoking^13^), comorbid conditions (diabetes,^10,14,15^ kidney dysfunction,^13^ baseline neuropathy,^4,15,16,17,18^ and vitamin D deficiency^19^), and genetic factors.^20^ Treatment-related factors include a cumulative taxane dose, the frequency of dosing, and the type of taxane.^10,12,21,22^ However, results of prior studies have been inconsistent, as many of these studies have been retrospective, included small sample sizes, or varied in the measures used to assess and define neuropathy.

SWOG S1714 is a prospective observational cohort study of TIPN in participants with nonmetastatic cancer receiving a taxane-based chemotherapy regimen. Over 3 years, participants were assessed for neuropathy symptoms using patient-reported outcome measures, clinician-assessed measures, and objective neurosensory and functional testing. The primary objective was to develop and validate a TIPN risk prediction model.

## Methods

### Study Design and Participants

S1714 (ClinicalTrials.gov: NCT03939481) enrolled participants 18 years or older with stage I to III non–small cell lung cancer or breast, ovarian, fallopian tube, or primary peritoneal cancer who started taxane-based treatment between March 1, 2019, and November 15, 2021, with 3 years of follow-up. Eligible taxane-based regimens were protocol specified and included either paclitaxel or docetaxel (eTable 1 in Supplement 1). The current study received ethical review and approval by the National Cancer Institute (NCI) Central Institutional Review Board, in compliance with the provisions of the Declaration of Helsinki^23^ and Good Clinical Practice guidelines, and participants provided written informed consent. This study followed the Strengthening the Reporting of Observational Studies in Epidemiology (STROBE) reporting guideline and is adherent to the Transparent Reporting of a Multivariable Prediction Model for Individual Prognosis or Diagnosis (TRIPOD) reporting guideline statement.

### Procedures

At registration, information on self-reported demographic characteristics (age, sex, and race and ethnicity); oncologic history (primary malignant neoplasm and stage); planned taxane treatment (paclitaxel or docetaxel), frequency (weekly, biweekly, or triweekly), dosing (full or reduced), and duration; planned use of platinum agent (yes or no); baseline comorbid conditions; creatinine clearance; Zubrod performance status; history of falls within past 6 months; and smoking history was collected (Table). Serial assessments of TIPN were conducted at registration (prior to initiation of taxane) and at 4, 8, 12, and 24 weeks using the patient-reported European Organization for Research and Treatment of Cancer Quality of Life Questionnaire–Chemotherapy-Induced Peripheral Neuropathy 20-item scale (CIPN-20).^24^ Assessments of TIPN continued through weeks 52, 104, and 156 (not included in current analyses), and biological samples were collected for future correlative analyses. Standardized trial data collection, monitoring, and quality checks were performed uniformly across all participants.

**Table.** Baseline Characteristics of Evaluable Patients

Characteristic	Patients, No. (%)		
	Total (N = 1278)	Training set (n = 768)	Test set (n = 510)
**Demographic characteristics**			
Age, median (range), y	55.0 (23.0-84.0)	55.0 (23.0-84.0)	56.0 (27.0-83.0)
Sex			
Male	14 (1.1)	9 (1.2)	5 (1.0)
Female	1264 (98.9)	759 (98.8)	505 (99.0)
Race			
Asian	56 (4.4)	35 (4.6)	21 (4.1)
Black	142 (11.1)	69 (9.0)	73 (14.3)
Native American	4 (0.3)	1 (0.1)	3 (0.6)
Pacific Islander	19 (1.5)	11 (1.4)	8 (1.6)
White	944 (73.9)	583 (75.9)	361 (70.8)
Multiple races	10 (0.8)	9 (1.2)	1 (0.2)
Unknown	103 (8.1)	60 (7.8)	43 (8.4)
Ethnicity			
Hispanic or Latino	136 (10.6)	82 (10.7)	54 (10.6)
Non-Hispanic or non-Latino	1132 (88.6)	680 (88.5)	452 (88.6)
Unknown	10 (0.8)	6 (0.8)	4 (0.8)
**Baseline comorbidities**			
History of falls within last 6 mo			
Yes	121 (9.5)	70 (9.1)	51 (10.0)
No	1157 (90.5)	698 (90.9)	459 (90.0)
Baseline conditions			
Diabetes	187 (14.6)	111 (14.5)	76 (14.9)
Thyroid disease	189 (14.8)	126 (16.4)	63 (12.4)
Vitamin B_12_ supplementation	28 (2.2)	11 (1.4)	17 (3.3)
Autoimmune disease	60 (4.7)	37 (4.8)	23 (4.5)
Vitamin D supplementation	122 (9.5)	73 (9.5)	49 (9.6)
Neurologic condition	66 (5.2)	46 (6.0)	20 (3.9)
Moderate kidney disease (creatinine clearance ≤44 mL/min)	18 (1.4)	16 (2.1)	2 (0.4)
Smoking history			
Never	819 (64.1)	500 (65.1)	319 (62.5)
Former	275 (21.5)	154 (20.1)	121 (23.7)
Active	182 (14.2)	112 (14.6)	70 (13.7)
Unknown	2 (0.2)	2 (0.3)	0
Baseline CIPN-20 sensory subscale score, mean (SD)	5.5 (10.2)	5.4 (10.3)	5.8 (10.0)
Zubrod performance status, No. (%)			
0	954 (74.6)	562 (73.2)	392 (76.9)
≥1	324 (25.4)	206 (26.8)	118 (23.1)
**Oncologic and treatment factors**			
Malignant neoplasm			
Breast	1164 (91.1)	697 (90.8)	467 (91.6)
Lung	9 (0.7)	5 (0.7)	4 (0.8)
Ovarian, fallopian tube, or peritoneal	105 (8.2)	66 (8.6)	39 (7.6)
Stage			
I	511 (40.0)	314 (40.9)	197 (38.6)
II	499 (39.0)	295 (38.4)	204 (40.0)
III	265 (20.7)	157 (20.4)	108 (21.2)
Taxane			
Paclitaxel	767 (60.0)	461 (60.0)	306 (60.0)
Docetaxel	511 (40.0)	307 (40.0)	204 (40.0)
Planned frequency			
Weekly	601 (47.0)	362 (47.1)	239 (46.9)
Biweekly	54 (4.2)	29 (3.8)	25 (4.9)
Triweekly	623 (48.7)	377 (49.1)	246 (48.2)
Planned taxane dosing			
Full dose	1259 (98.5)	757 (98.6)	502 (98.4)
Reduced dose	19 (1.5)	11 (1.4)	8 (1.6)
Planned use of platinum agent			
Yes	414 (32.4)	248 (32.3)	344 (67.5)
No	864 (67.6)	520 (67.7)	166 (32.5)
Planned duration of taxane, wk			
≤12	961 (75.2)	570 (74.2)	391 (76.7)
>12	317 (24.8)	198 (25.8)	119 (23.3)

Abbreviation: CIPN-20, European Organization for Research and Treatment of Cancer Quality of Life Questionnaire–Chemotherapy-Induced Peripheral Neuropathy 20-item scale.

### Primary End Point

The primary end point, development of TIPN, was defined using the CIPN-20.^24^ Prior studies indicated that patients with chemotherapy-induced peripheral neuropathy experience approximately a 7- to 10-point increase in the CIPN-20 sensory neuropathy subscale score.^25,26,27^ Based on these considerations, participants having an absolute increase of 8 points or more over the baseline sensory neuropathy subscale score by 24 weeks were defined as having experienced TIPN. This difference would correspond, for instance, to 1 symptom (eg, tingling) worsening by 2 response categories (eg, from a little to very much) or 2 items worsening by 1 response category.

### Statistical Analysis

Statistical analysis was conducted from December 2023 to June 2024 with SAS, version 9.4 (SAS Institute Inc), and R, version 4.3.2 (R Project for Statistical Computing). A TIPN risk prediction model was derived in 2 steps. First, 60% of evaluable participants (stratified by taxane regimen) were randomly sampled to build the model. Potential risk factors (Table) were ranked in univariate models using χ^2^ statistics (eMethods in Supplement 1). Model building was based on logistic regression to simplify identification and interpretation of adverse risk for each factor. Best-subset selection identified the best *k*-variable model from among the factors most predictive of TIPN in univariate examinations.^28^ The goal was to minimize predictive error (ie, logistic model deviance) across various models. The *k*-fold cross-validation of the entire model-building approach, including the identification of univariate predictors for inclusion in best-subset selection analysis, was used to limit overfitting and provide insight into how the model will generalize in step 2.^29^ Once the best model was identified, a risk model was built by summing the number of adverse risk factors from among the *k* variables to generate a score, split at the median, to identify participants at high vs low risk.

In step 2, the risk model from step 1 was tested for the remaining 40% of evaluable participants. The primary aim was to identify an absolute 12% difference in risk of TIPN between the high-risk vs low-risk groups, based on a predicted overall TIPN rate of 30%.

The study initially targeted 1050 participants (525 each for paclitaxel and docetaxel). Given rapid accrual and a 3:2 enrollment imbalance favoring paclitaxel, the target was increased to 1310 participants to achieve 1245 eligible participants, ensuring 500 participants or more per group. In step 1, 60% of evaluable participants (n = 745) provided 85% power or more to detect an absolute 10% difference in TIPN rates between the low-risk and high-risk groups (≤30% overall incidence) using 2-sided α = .05 binomial tests. In step 2, the remaining 40% (n = 500) provided greater than 90% power to identify a 12% absolute difference using a 1-sided α = .05 test; failure to detect this difference would indicate limited ability to discriminate TIPN risk based on clinical factors alone. In addition, the C statistic was calculated, and the dose-response association was assessed by evaluating TIPN risk according to the quartile distribution of the adverse risk score.^30^ Model calibration in the validation set was evaluated by plotting observed vs predicted event rates across deciles of predicted probability and by estimating the calibration intercept and slope. Unless otherwise specified, all *P* values were from 2-sided tests and results were deemed statistically significant at *P* < .05.

## Results

### Accrual and Demographics

**Figure 1.**
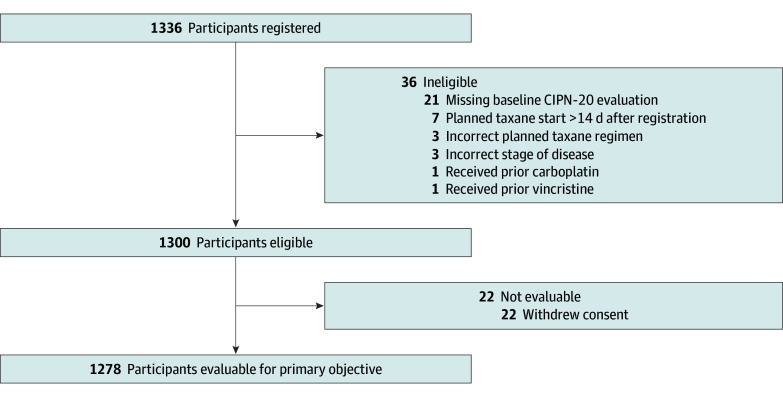
Flow Diagram of Participants in S1714 Study CIPN-20 indicates European Organization for Research and Treatment of Cancer Quality of Life Questionnaire–Chemotherapy-Induced Peripheral Neuropathy 20-item scale.

The study was open to accrual between March 1, 2019, and November 15, 2021, at 105 sites in the NCI National Community Oncology Research Program. In total, 1336 participants were registered, and 1278 (median age, 55.0 years [range, 23.0-84.0 years]; 1264 women [98.9%] and 14 men [1.1%]; 56 Asian [4.4%], 142 Black [11.1%], 136 Hispanic [10.6%], 4 Native American [0.3%], 19 Pacific Islander [1.5%], 944 White [73.9%], and 10 multiracial [0.8%]) were evaluable (Table and Figure 1). Baseline characteristics are shown in the Table. The mean follow-up time was 168 days (90th percentile IQR, 84-168 days). Factors were well balanced between the training and test sets.

### Baseline Comorbid Conditions

The most common comorbid conditions included thyroid disease (189 participants [14.8%]) and diabetes (187 participants [14.6%]) (Table). There was minimal sensory neuropathy at baseline as measured by the CIPN-20 sensory subscale.

### Oncologic and Treatment Characteristics

Most participants had a diagnosis of breast cancer (1164 [91.1%]) (Table). Diagnosis by stage included 511 participants (40.0%) with stage I disease, 499 (39.0%) with stage II disease, and 265 (20.7%) with stage III disease. A total of 767 participants (60.0%) were planned to be treated with paclitaxel and 511 (40.0%) with docetaxel. Almost all participants (1259 [98.5%]) planned to receive the taxane at full dose. The most common planned frequency of taxane dosing was triweekly (623 [48.7%]), followed by weekly (601 [47.0%]) and biweekly (54 [4.2%]). Taxane duration of 12 weeks or less was planned for 961 participants (75.2%). Administration of a concurrent platinum agent was planned for 414 participants (32.4%).

### Risk Model Development

By week 24, 804 evaluable participants (62.9%) experienced TIPN. Using a training set of 768 participants, a risk prediction model for TIPN was developed that included 5 risk factors (eMethods and eFigure 1 in Supplement 1): receipt of paclitaxel; stage II or III cancer diagnosis; planned duration of taxane treatment of more than 12 weeks; having a diagnosis of diabetes, autoimmune disease, moderate kidney disease (creatinine clearance ≤44 mL/min), and/or a neurologic condition; and self-identification as Black, Hispanic, Native American, Pacific Islander, multiple races, or unknown race or ethnicity. The median adverse risk score was 2 (range, 0-5), with low risk defined as 1 risk factor or less (n = 259 [33.7%]) and high risk as 2 risk factors or more (n = 509 [66.3%]). TIPN occurred in 125 of 259 participants at low risk (48.3%) and 360 of 509 participants at high risk (70.7%) (odds ratio [OR], 2.59 [95% CI, 1.90-3.53]; *P* < .001). The C statistic for the 4-level model was 0.64 (95% CI, 0.60-0.68).

### Risk Model Validation

In the test set of 510 participants, 165 (32.4%) met low-risk criteria and 345 (67.6%) met high-risk criteria. TIPN occurred in 84 of 165 participants at low risk (50.9%) and 235 of 345 participants at high risk (68.1%) (OR, 2.06 [95% CI, 1.41-3.01]; *P* < .001). The absolute percentage difference between the high-risk and low-risk groups was 17.2%, which exceeded the protocol-specified target difference of 12%. The C statistic for the 4-level model was 0.62 (95% CI, 0.57-0.67). The calibration intercept was 0.05 (95% CI, −0.21 to 0.32), and the slope was 0.83 (95% CI, 0.62-1.03), consistent with overall good agreement between predicted and observed risk, albeit with a modest overestimation among patients at higher risk (eFigure 2 in Supplement 1).

### Application of Risk Model to Entire Cohort

**Figure 2.**
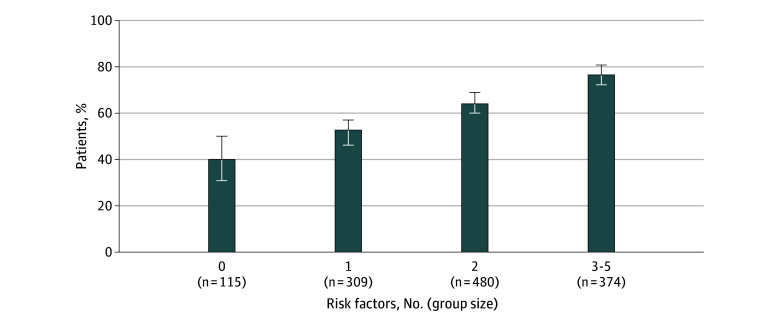
Bar Graph of Risk of Taxane-Induced Peripheral Neuropathy by Number of Risk Factors in Total Evaluable Cohort Level-specific binomial 95% CIs are provided.

**Figure 3.**
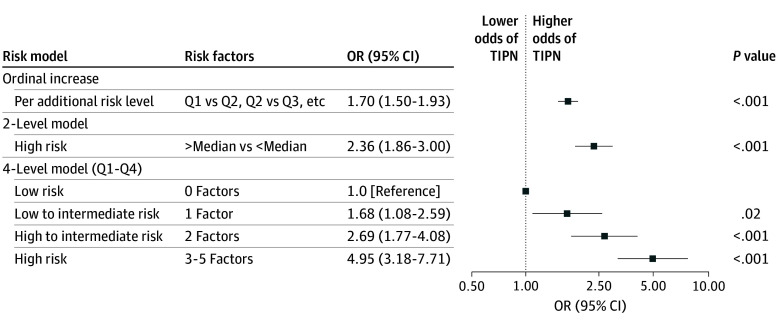
Forest Plot of the Odds of Taxane-Induced Peripheral Neuropathy (TIPN) Based on Risk Model Each box indicates the odds ratio (OR) and the horizontal lines show the 95% CIs. The line at 1.00 indicates the line of equal odds (ie, no difference).

Among all evaluable participants (N = 1278), the risk of TIPN increased substantially as quartile-level risk increased (Figure 2), from 40.0% (46 of 115) for those in the lowest quartile (0 risk factors) to 76.7% (287 of 374) for those in the highest quartile (3-5 risk factors), for a 36.7% difference between the highest and lowest quartiles. Figure 3 shows a forest plot of the odds of TIPN based on ordinal increase, 2-level model split at median, and 4-level model by quartiles. For each increase in quartile risk level, the odds of TIPN increased 70% (OR, 1.70 [95% CI, 1.50-1.93]; *P* < .001). When split at the median level, participants with 2 or more risk factors had a greater than 2-fold increase in odds of TIPN (OR, 2.36 [95% CI, 1.86-3.00]; *P* < .001). There was a nearly 5-fold increased risk of TIPN for those in the highest quartile (3-5 risk factors) vs the lowest quartile (0 risk factors) (OR, 4.95 [95% CI, 3.18-7.71]; *P* < .001). The C statistic for the 4-level risk prediction model was 0.63 (95% CI, 0.60-0.66). The association of model factors with TIPN in a multivariable model is shown in eTable 2 in Supplement 1, and the distribution of the risk factors by risk factor groups is shown in eTable 3 in Supplement 1.

In a post hoc analysis that excluded the race and ethnicity risk factor, the C statistic for the 4-level risk prediction model was 0.62 (95% CI, 0.59-0.65) (eTable 4 in Supplement 1). The primary model results were robust to different weighting strategies for the factors included in the model (eTable 5 in Supplement 1).

## Discussion

In a large, prospective risk prediction cohort study, we developed and validated a TIPN risk prediction model based on 5 clinical factors that can be readily identified. Based on this model, the study met its predefined study end point, differentiating a 17.2% difference in TIPN between those at low risk and those at high risk. The model was able to differentiate nearly a 5-fold increase in risk of TIPN between those with 3 to 5 risk factors (highest quartile) and those with 0 risk factors (lowest quartile).

The inclusion of paclitaxel as a risk factor for TIPN is consistent with findings from several prior studies demonstrating an increase in neuropathy with the use of paclitaxel compared with docetaxel. Use of nab-paclitaxel was not allowed in this trial due to the known increased risk of TIPN.^21^ In the E1199 trial evaluating the efficacy of paclitaxel vs docetaxel, patients receiving weekly paclitaxel for 12 cycles had a higher incidence of Common Terminology Criteria for Adverse Events (CTCAE) grade 2 to 4 neuropathy (27%) than patients receiving paclitaxel every 3 weeks for 4 cycles (20%), weekly docetaxel for 12 cycles (16%), or docetaxel every 3 weeks for 4 cycles (16%).^22^ Data from the EAZ171 trial, which specifically enrolled Black women receiving taxane-based chemotherapy, also demonstrated higher rates of CTCAE-assessed TIPN for weekly paclitaxel compared with docetaxel every 3 weeks (44% vs 25% for grade ≥2).^12^ Planned duration of taxane for more than 12 weeks was also identified as a risk factor. Higher cumulative dose is a known risk factor for TIPN.^31,32,33,34^

The presence of a diagnosis of diabetes, moderate kidney disease, autoimmune disease, and/or a neurologic condition at time of registration were also found to be risk factors for the development of TIPN. Prior studies have shown an association between a diagnosis of diabetes^10,14,15^ or decreased creatinine clearance^13^ and TIPN, consistent with our findings. There are conflicting data regarding autoimmune disease as a risk factor. A retrospective study of patients aged 65 years or older receiving taxane therapy suggested that patients with a diagnosis of autoimmune disease were less likely to have CTCAE grade 2 to 4 neuropathy compared with those without the diagnosis (OR, 0.49 [95% CI, 0.24-1.02]; *P* = .06), although power for this observation was limited and the difference was not statistically significant.^10^ Conversely, in a large population-based study of survivors of early-stage breast cancer, autoimmune disease was associated with increased risk of neuropathy symptoms.^35^ Further investigation is needed to better understand the association between autoimmune disease and TIPN. The diagnosis of a neurologic condition was also a risk factor for TIPN, with most described as “other neurological conditions.” Although this is a provocative finding, this study lacks granular details about the neurologic conditions to enable a conclusion about possible mechanisms.

There are several factors that may be associated with disease stage being a risk factor for TIPN. Those with higher-stage disease may be at increased risk of recurrence, and thus physicians and patients may be reluctant to reduce the dose of taxanes in curative-intent therapy. In addition, more than 90% of the participants included in the study had a diagnosis of breast cancer, and thus having stage II or III breast cancer likely reflects larger tumor size or axillary lymph node involvement, which may require mastectomy or axillary lymph node dissection. Exploratory analysis of the NRG/NSABP-30 trial found that mastectomy and greater number of positive lymph nodes were associated with higher risk of peripheral neuropathy.^34^ As such, postsurgical toxic effects may be associated with neuropathy among some individuals.

Racial and ethnic disparities in TIPN between individuals of Black or African ancestry and White individuals have been reported. Associated factors are complex,^36^ and may include differences in the social determinants of health, including structural disparities; completion and interpretation of patient-reported outcomes^37^; and vitamin D insufficiency.^19^ A correlative study of EA-5103 found that individuals with genetically determined African ancestry experienced higher TIPN rates measured by CTCAE compared with individuals of European ancestry (grades 2-4: OR, 2.2 [*P* < .001]; grades 3-4: OR, 2.9 [*P* < .001]).^9^ This increase in TIPN ultimately led to more paclitaxel dose reductions among those of African ancestry, negatively affecting disease-free survival.^9^ Although germline biomarkers for racial disparities in TIPN have been hypothesized, in EAZ171, variants in 2 genes (*FCAMR* and *SBF2*) were not significantly associated with increased TIPN rates.^12^ Data on TIPN disparities among other racial and ethnic groups, such as Hispanic, Native American, Pacific Islander, and multiracial individuals, are limited. Due to potential challenges in the interpretation of self-identified race and ethnicity, including an unknown race category, we excluded race and ethnicity as a variable in a post hoc analysis; the model had similar performance (C statistic of 0.63 with race and ethnicity risk factor and 0.62 without race and ethnicity risk factor).

There are several previously described risk factors for neuropathy assessed in this trial that were not included in the model. Approximately one-third of participants in the study received a taxane with a concurrent platinum agent, another chemotherapy with known neurotoxic effects,^10,14,38^ but this was not associated with increased risk. Increasing age^10^ and smoking history^13^ have also been previously reported as risk factors for TIPN but did not meet criteria for inclusion in the model. The presence of baseline neuropathy has been reported as a risk factor for TIPN.^4,15,16,17,18^ Unlike prior studies that used an absolute threshold, such as CTCAE grade 2 or higher, to define TIPN, S1714 defined TIPN as a change in the CIPN-20 sensory score from baseline. The differences in identified risk factors may be due to variability in the definition of TIPN, the study population, or other complex interactions between potential risk factors, including the possibility that our comprehensive multivariable evaluation of a range of factors may have adequately accounted for confounding, obviating some previously observed associations.

### Strengths and Limitations

This study has some strengths. The prospective risk prediction design overcomes limitations of prior studies aimed at characterizing the phenotype of TIPN, especially those based on retrospective data collection subject to ascertainment bias, recall bias, and missing data. The model was trained and tested in independent datasets, with type I error accounted for through the validation step. The study population included a large, racially and ethnically diverse sample of participants treated in the community oncology setting, representative of the setting in which most patients with cancer are treated. Also, the study used a prospectively collected patient-reported outcome measure, the CIPN-20, as the primary end point measure, which is likely a better estimate of symptoms compared with clinician assessments of neuropathy, such as the CTCAE.^39,40,41^

This study also has some limitations. First, these data generalize best to female patients with breast cancer. Second, limited data were available to guide the assessment of a clinically meaningful difference in TIPN rates based on baseline clinical factors alone at the time of trial design. Our protocol-specified target difference was generally consistent with designs of randomized clinical trials for the prevention of TIPN, for which more conservative target differences in designs reflect the challenges of identifying effective therapeutic approaches for this condition.^42,43,44,45^ We identified a 17.2% difference between high-risk and low-risk groups in the test set. This threshold difference may not be sufficient to influence treatment decisions for some patients and their physicians, although any decision will ultimately be determined by a patient’s unique set of conditions, risks, and treatment requirements.^46^ Even with none of the 5 risk factors, the occurrence of TIPN was 40.0%. Given the modest C statistic (0.63), further model refinement, including incorporation of biomarkers, is ongoing to improve risk discrimination among taxane-treated patients. Third, while a simple count of risk factors does not reflect differences in individual variable contributions, it offers a readily interpretable and clinically usable approach; more complex weighting strategies provided minimal or no improvement in model performance.

## Conclusions

In this large prospective cohort study of participants with early-stage cancer receiving a taxane-based regimen, the rate of TIPN by 24 weeks was 62.9%. With its innovative design, S1714 prospectively evaluated numerous prespecified potential risk factors simultaneously to develop and validate a risk model identifying the combination of factors most relevant in determining risk of TIPN. In addition, this risk model can be easily applied in the oncology clinic. The large difference (36.7%) between those in the highest quartile (3-5 risk factors) and those in the lowest quartile (0 risk factors) may help guide decision-making when other options are available. Enhancing the ability to predict the risk of TIPN may influence physician monitoring for mitigation strategies and prioritize enrollment in clinical trials investigating interventions for TIPN prevention and treatment.

## Notes

Supplement 1.
eMethods.
**eTable 1.** Taxane-Based Regimens Included in S1714 **eTable 2.** Association of Model Factors With TIPN in a Multivariable Logistic Regression Model Among All Patients **eTable 3.** Distribution of Risk Factors by Risk Factor Groups (in Quartiles) **eTable 4.** Odds of Taxane-Induced Peripheral Neuropathy (TIPN) Based on the Risk Model in a Post Hoc Analysis Excluding the Race/Ethnicity Variable **eTable 5.** Primary Model Results for Different Approaches to Weighting Model Covariates **eFigure 1.** Relative Change by Number of Model Variables **eFigure 2.** Calibration Plot

Supplement 2.
Data Sharing Statement
