# Supplementary material for: Risk Prediction Model for Taxane-Induced Peripheral Neuropathy in Early-Stage Cancer
Source: JAMA Netw Open. 2026 Apr 10;9(4):e264901. doi: 10.1001/jamanetworkopen.2026.4901 (PMC13069458; doi:10.1001/jamanetworkopen.2026.4901)
Supplement: Supplement 1. — eMethods. eTable 1. Taxane-Based Regimens Included in S1714 eTable 2. Association of Model Factors With TIPN in a Multivariable Logistic Regression Model Among All Patients eTable 3. Distribution of Risk Factors by Risk Factor Groups (in Quartiles) eTable 4. Odds of Taxane-Induced Peripheral Neuropathy (TIPN) Based on the Risk Model in a Post Hoc Analysis Excluding the Race/Ethnicity Variable eTable 5. Primary Model Results for Different Approaches to Weighting Model Covariates eFigure 1. Relative Change by Number of Model Variables eFigure 2. Calibration Plot [file jamanetwopen-e264901-s001.pdf]

Supplementary Online Content

Trivedi MS, Unger JM, Henry NL, et al. Risk prediction model for taxane-induced peripheral neuropathy in early-stage cancer. *JAMA Netw Open.* 2026;9(4):e264901. doi:10.1001/jamanetworkopen.2026.4901

eMethods.

eTable 1. Taxane-Based Regimens Included in S1714

eTable 2. Association of Model Factors With TIPN in a Multivariable Logistic Regression Model Among All Patients

eTable 3. Distribution of Risk Factors by Risk Factor Groups (in Quartiles)

eTable 4. Odds of Taxane-Induced Peripheral Neuropathy (TIPN) Based on the Risk Model in a Post Hoc Analysis Excluding the Race/Ethnicity Variable

eTable 5. Primary Model Results for Different Approaches to Weighting Model Covariates

eFigure 1. Relative Change by Number of Model Variables

eFigure 2. Calibration Plot

This supplementary material has been provided by the authors to give readers additional information about their work.

eMethods.

Summary of Model Training Procedures and Results for S1714, "A Prospective Observational Cohort Study to Develop a Predictive Model of Taxane-Induced Peripheral Neuropathy in Cancer Patients"

Among the N=1336 enrolled participants, 1300 were eligible and 1278 were evaluable [**Figure 1**]. Per protocol, from among these n=1278 eligible and evaluable participants, we drew a random 60% subsample of 768 participants to serve as the training set. Sample characteristics are shown in **Table 1** in the main text.

In exploratory (i.e., training) evaluations of the association of the individual factors and any TIPN occurrence by week 24, the following variables were considered as candidate risk factors for predicting TIPN occurrence by week 24. In some instances, variables were recoded according to observed patterns. In general, we aimed to dichotomize continuous variables to aid interpretation, especially when aligned with known constructs.

<u>Demographics</u>

- Age: Age was dichotomized as <65 vs 65 or older based on known associations with social insurance programs (e.g., Social Security, Medicare) related to access to economic and healthcare resources.
- Sex: Sex was coded as male vs. female.
- Race/ethnicity: TIPN proportions were notably higher among Black (78%), Native American (100%), Pacific Islander (82%), Multiple (78%) and Unknown (70%) race participants compared to Asian (63%) and White participants (60%). TIPN was also higher among Hispanic (71%) compared to non-Hispanic (62%) participants. Based on these observed patterns, an aggregate race/ethnicity variable was created as Black, Native American, Pacific Islander, multiple or unknown race or Hispanic ethnicity participants vs. non-Hispanic Asian and White participants.

<u>Baseline Comorbidities</u>

- History of Falls within Last 6 Months: Coded yes vs. no.
- Baseline Conditions: The evaluation of baseline comorbid conditions was limited by sample size considerations. For instance, almost no participants (n=3) had baseline creatinine clearance <30 mL/min. Instead, we used the baseline creatinine clearance level of 44 mL/min to categorize those with moderate kidney disease (yes vs. no); using this cutoff, observed TIPN proportions among the 16 participants with moderate kidney disease or worse was 75% compared to 63% for other participants. Other baseline conditions for which the observed proportions were >=5% larger (in absolute percentages) compared to those without the condition included diabetes (70% vs. 62%), neurologic condition (70% vs. 63%), and autoimmune disease (68% vs. 63%), but not for other comorbid conditions. Thus, we dichotomized the number of baseline conditions as 0 vs. 1 or more of baseline diabetes, moderate kidney disease, neurologic condition, and/or autoimmune disease.
- Smoking History: To limit model complexity, we categorized smoking status as never smoker vs. ever smoker.
- Baseline CIPN-20 sensory subscale score: Various levels of the baseline CIPN-20 sensory subscale score were explored, with limited or no evidence of an association with TIPN within 24 weeks after enrollment. For model evaluation, we categorized the sensory subscale score as presence vs. absence of TIPN at baseline at the protocol-specified level of 8 points, consistent with the TIPN endpoint definition.
- Performance Status: Given the few participants with performance status >1, performance status was categorized as 0 vs. >=1.

<u>Oncologic and Treatment Factors</u>

- Malignant neoplasm: Few enrolled participants in the study had lung cancer. Based on observed proportions, we categorized malignant neoplasm as breast cancer vs. other.
- Stage: TIPN was strongly associated with the prognostic stage, with TIPN proportions of 56.1%, 67.1%, and 70.1% for stages I, II, and III, respectively (p=.002). Based on these observed percentages, we categorized stage as an indicator variable (stage II or III vs. stage I).
- Taxane Treatment: Planned treatment was coded as paclitaxel vs. docetaxel.
- Planned Frequency: Planned frequency of taxane treatment administration was categorized as weekly (66% with TIPN) vs. biweekly (69% with TIPN) vs. triweekly (60% with TIPN). Frequency was included in the regression models using 2 separate indicator variables, with weekly administration as the reference group.

© 2026 Trivedi MS et al. *JAMA Network Open.*

- Planned Taxane Dosing: Planned taxane dosing was categorized as full vs. reduced dose.
- Planned Use of a Platinum Agent: Categorized as yes vs. no.
- Planned Duration of Taxane: Based on the observation of widely varying TIPN rates according to disease stage, and the concern that stage may be encoding for duration of planned taxane-based therapy, we also included planned treatment duration as a covariate in the training evaluation, categorized as >12 weeks vs. <=12 weeks.

All predictors were coded as binary indicators (e.g., presence (1) versus absence (0) of event). Absence included instances of non-reporting, which was assumed to reflect non-occurrence given standardized clinical trial data collection.

As noted in the Methods section, these potential factors considered for inclusion in the TIPN risk prediction model were evaluated in univariate models and ranked according to their chi-square statistic using logistic regression.

We used the method of best subsets to identify a ***q***-variable model that minimized mean squared error based on the Akaike information criterion (AIC). Of note, individual indicators included in composite variables for comorbid conditions and race/ethnicity were not separately included in the best subsets regression model. Thus in total, 19 variables were included in the best subsets regression analyses.

K-fold cross-validation was used. Because model statistics can vary according to the number of folds, we applied each of 2- and 3-fold validation strategies, each separately 20 times to account for variation in random draws. Additionally, model statistics can also vary according to the number of variables specified in the best subsets regression model. We allowed the number of model variables to increase from 12 to the maximum of 19 in units of 1 variable (total = 8 iterations) for each fold and random draw combination, representing a total of 2*20*8=320 models. The Supplemental **eFigure 1** below illustrates how the average AIC across the 2-fold (red) and 3-fold (blue) cross-validation strategies identified a 5-variable model as the model that minimized the AIC. In the entire training set, the 5 adverse risk factors were receipt of receipt of paclitaxel; Stage II or III cancer diagnosis; planned duration of taxane > 12 weeks; having a diagnosis of diabetes, autoimmune disease, moderate kidney disease (CrCl < 44), and/or a neurologic condition; and self-identification as Black, Native American, Pacific Islander, or Multiple or Unknown race or self-identification as Hispanic ethnicity.

© 2026 Trivedi MS et al. *JAMA Network Open.*

eTable 1. Taxane-Based Regimens Included in S1714

**Breast cancer**		
**Regimen**	**Standard Taxane Dose**	**Frequency**
*P x 4	Paclitaxel 175 mg/m2	Every 2 weeks for 4 cycles
*P x 12 +/- Carboplatin (AUC 2 for weekly or AUC 5-6 for every 3 weeks)	Paclitaxel 80 mg/m2	Every week for 12 weeks (1 week = 1 cycle)
TC x 4 or TC x 6	Docetaxel 75 mg/m2	Every 3 weeks for 4 or 6 cycles
TAC x 6	Docetaxel 75 mg/m2	Every 3 weeks for 6 cycles
*T x 4	Docetaxel 100 mg/m2	Every 3 weeks for 4 cycles
T/Carboplatin (AUC 6)	Docetaxel 75 mg/m2	Every 3 weeks for 6 cycles
**Lung cancer**		
**Regimen**	**Standard Taxane Dose**	**Frequency**
Carboplatin (AUC 6) + P x 4	Paclitaxel 200 mg/m2	Every 3 weeks for 4 cycles
**Ovarian/Fallopian tube/ Peritoneal cancer**		
**Regimen**	**Standard Taxane Dose**	**Frequency**
Carboplatin (AUC 5-6) + P x 6	Paclitaxel 175 mg/m2	Every 3 weeks for 6 cycles
Carboplatin (AUC 5-6) + ddP x 6	Paclitaxel 80 mg/m2	Carboplatin every 3 weeks and paclitaxel weekly for 18 weeks
Carboplatin (AUC 2) + P x 18	Paclitaxel 60 mg/m2	Weekly for 18 weeks
Carboplatin (AUC 5-6) + T x 6	Docetaxel 60-75 mg/m2	Every 3 weeks for 6 cycles
Abbreviations: dd, dose-dense; A, doxorubicin; C, cyclophosphamide; P, paclitaxel; T, docetaxel<br>Biologic agents, including but not limited to trastuzumab and/or pertuzumab, may be added to any of the regimens.<br>* Can be preceded or followed by AC.		

© 2026 Trivedi MS et al. *JAMA Network Open.*

eTable 2. Association of Model Factors With TIPN in a Multivariable Logistic Regression Model Among All Patients

**Model Variables**	**OR**	**95% CI**	**P-value**
Receipt of Paclitaxel (vs. docetaxel)	2.15	1.66-2.78	<.001
Stage 2 or 3 (vs. grade 1)	1.33	1.04-1.70	.02
Planned taxane duration > 12 weeks (vs. <=12 weeks)	1.72	1.28-2.31	<.001
Black, Native American, Pacific Islander, Multiple, or Unknown race or Hispanic ethnicity (vs. White or Asian race)	1.69	1.27-2.25	<.001
Diagnosis of diabetes, autoimmune disease, moderate kidney disease, and/or neurologic condition (vs. none)	1.54	1.15-2.05	.003

© 2026 Trivedi MS et al. *JAMA Network Open.*

eTable 3. Distribution of Risk Factors by Risk Factor Groups (in Quartiles)

	Risk Quartile				
	**Q1: 0 RFs (N=115)**	**Q2: 1 RFs (N=309)**	**Q3: 2 RFs (N=480)**	**Q4: 3-5 RFs (N=374)**	**Overall (N=1278)**
Paclitaxel	0	162 (52.4)	316 (65.8)	289 (77.3)	767 (60.0)
Stage 2/3 disease	0	43 (13.9)	373 (77.7)	348 (93.0)	764 (59.8)
Planned taxane duration >12 weeks	0	47 (15.2)	108 (22.5)	162 (43.3)	317 (24.8)
Diabetes, autoimmune disease, moderate kidney disease, or a neurologic condition	0	35 (11.3)	84 (17.5)	177 (47.3)	296 (23.2)
Self-identified race/ethnicity of Black, Native American, Pacific Islander, Multiple, or Unknown race or Hispanic ethnicity	0	22 (7.1)	79 (16.5)	216 (57.8)	317 (24.8)

RFs = Risk Factors

© 2026 Trivedi MS et al. *JAMA Network Open.*

eTable 4. Odds of Taxane-Induced Peripheral Neuropathy (TIPN) Based on the Risk Model in a Post Hoc Analysis Excluding the Race/Ethnicity Variable

**Risk Model**	**Number or Risk Factors**	**OR (95% CI)**	**P-value**
Ordinal Increase			
Per additional risk level (Q1 vs. Q2, Q2 vs. Q3, etc)	1.67	1.56-1.91	<.001
2-Level Model			
High Risk (>Median vs. <Median)	2.23	1.77-2.82	<.001
4-Level Model (Quartiles)			
Low Risk (0 factors)	Reference		
Low-Intermediate Risk (1 factor)	1.88	1.26-2.80	.002
High-Intermediate Risk (2 factors)	3.25	2.21-4.78	<.001
High Risk (3-4 factors)	4.50	2.83-7.16	<.001

© 2026 Trivedi MS et al. *JAMA Network Open.*

eTable 5. Primary Model Results for Different Approaches to Weighting Model Covariates

	**Model Specifications**				
	Percentiles (0.01)	Deciles (0.10)	Quartiles (0.25)	Quantiles (0.50)	Equal weights[1]
**Training Set**					
Percentage TIPN					
Low risk group	53.5	49.0	54.9	48.0	48.3
High risk group	75.6	71.9	76.4	71.8	70.7
Absolute difference	22.1	22.9	21.5	23.8	22.4
Odds ratio<br>95% CI<br>p-value	2.70<br>1.97-3.68<br><.001	2.67<br>1.97-3.62<br><.001	2.66<br>1.92-3.68<br><.001	2.75<br>2.03-3.74<br><.001	2.59<br>1.90-3.53<br><.001
C-statistic	0.65	0.64	0.64	0.65	0.64
**Validation Set**					
Percentage TIPN					
Low risk group	56.7	51.5	55.6	51.7	50.9
High risk group	70.8	69.4	74.5	68.2	68.1
Absolute difference	14.1	17.9	18.9	16.5	17.2
Odds ratio<br>95% CI<br>p-value	1.85<br>1.27-2.69<br>.001	2.14<br>1.48-3.09<br><.001	2.33<br>1.57-3.46<br><.001	2.00<br>1.37-2.91<br><.001	2.06<br>1.41-3.01<br><.001
C-statistic	0.61	0.63	0.62	0.61	0.62
**All Patients**					
Percentage TIPN					
Low risk group	54.8	50.0	55.2	49.4	49.3
High risk group	73.7	70.9	75.7	70.3	67.7
Absolute difference	18.9	20.9	20.5	20.9	18.4
Odds ratio<br>95% CI<br>p-value	2.32<br>1.82-2.94<br><.001	2.44<br>1.93-3.09<br><.001	2.52<br>1.96-3.24<br><.001	2.42<br>1.91-3.07<br><.001	2.36<br>1.86-3.00<br><.001
C-statistic	0.63	0.63	0.63	0.63	0.63

[1] Protocol-specified model

© 2026 Trivedi MS et al. *JAMA Network Open.*

eFigure 1. Relative Change by Number of Model Variables

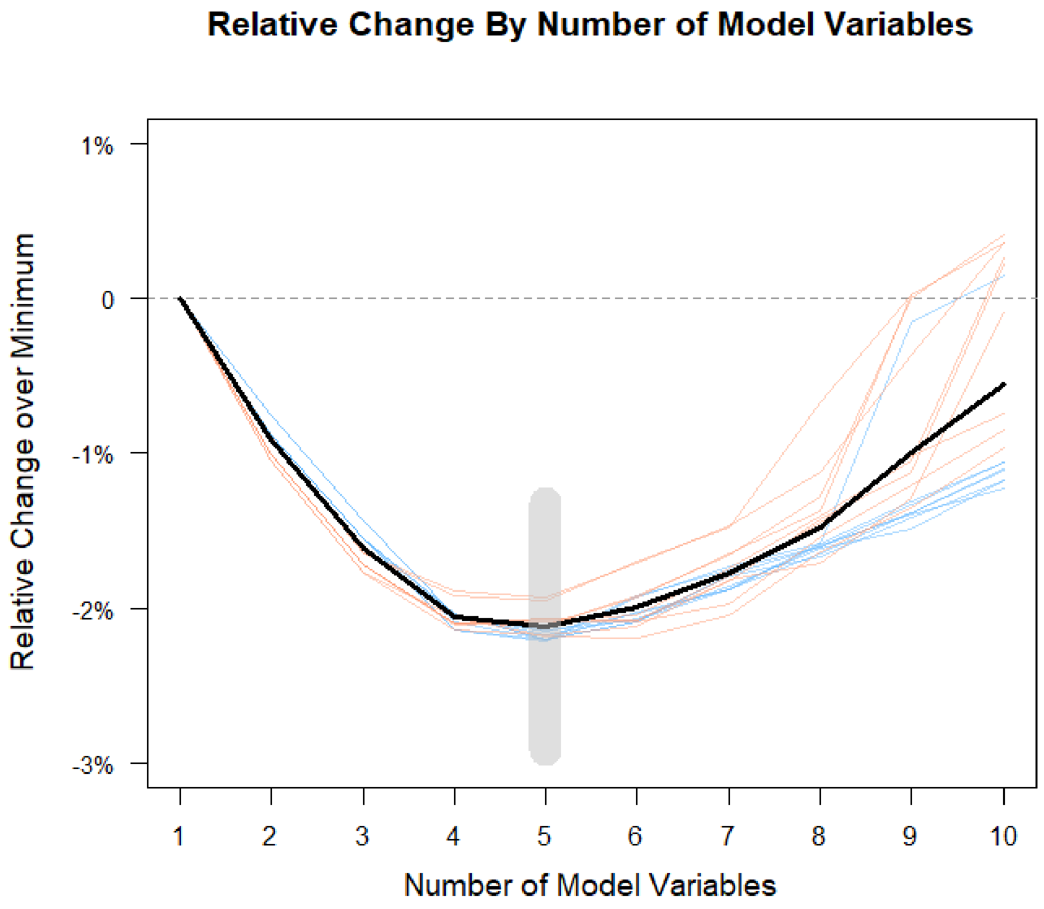

© 2026 Trivedi MS et al. *JAMA Network Open.*

eFigure 2. Calibration Plot.
A LOESS-smoothed calibration curve is shown.

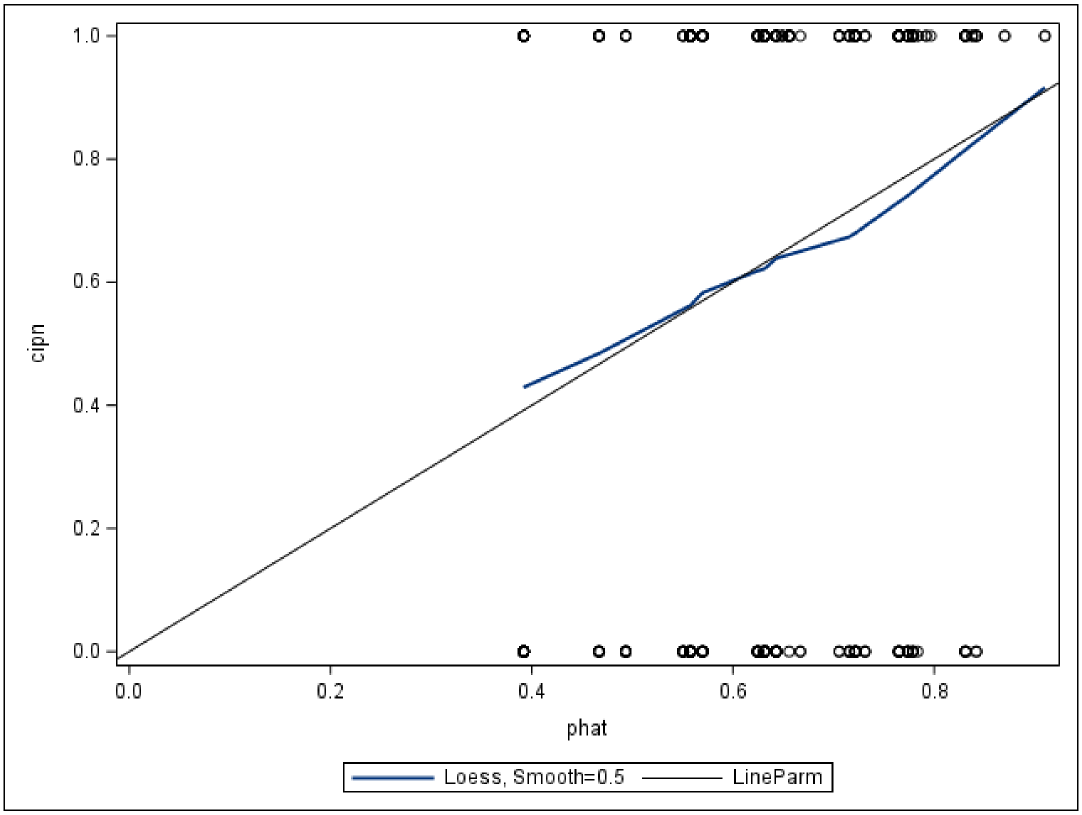

© 2026 Trivedi MS et al. *JAMA Network Open.*
