# Supplementary material for: Risk Prediction Model for Taxane-Induced Peripheral Neuropathy in Early-Stage Cancer
Source: JAMA Netw Open. 2026 Apr 10;9(4):e264901. doi: 10.1001/jamanetworkopen.2026.4901 (PMC13069458; doi:10.1001/jamanetworkopen.2026.4901)
Supplement: Supplement 2. — Data Sharing Statement [file jamanetwopen-e264901-s002.pdf]

Data Sharing Statement

Trivedi. Risk Prediction Model for Taxane-Induced Peripheral Neuropathy in Early-Stage Cancer. *JAMA Netw Open*. Published April 10, 2026. doi:10.1001/jamanetworkopen.2026.4901

Data
Data available: Yes
Data types: Deidentified participant data, Data dictionary
How to access data: Data can be requested by emailing S1714@swog.org.
When available: With publication

Supporting Documents
Document types: None

Additional Information
Who can access the data: Data pertinent to this analysis will be made available upon request for researchers whose proposed use of the data has been approved by the SWOG Cancer Research Network.
Types of analyses: Any purpose approved by the SWOG Cancer Research Network.
Mechanisms of data availability: Data pertinent to this analysis will be made available upon request for researchers whose proposed use of the data has been approved by the SWOG Cancer Research Network.
